# Efficacy of the treatment with dapagliflozin and metformin compared to metformin monotherapy for weight loss in patients with class III obesity: a randomized controlled trial

**DOI:** 10.1186/s13063-020-4121-x

**Published:** 2020-02-14

**Authors:** Aldo Ferreira-Hermosillo, Mario Antonio Molina-Ayala, Diana Molina-Guerrero, Ana Pamela Garrido-Mendoza, Claudia Ramírez-Rentería, Victoria Mendoza-Zubieta, Etual Espinosa, Moisés Mercado

**Affiliations:** 1 Unidad de Investigación Médica en Enfermedades Endocrinas, Hospital de Especialidades Centro Médico Nacional Siglo XXI, Instituto Mexicano del Seguro Social, Cuauhtémoc 330, Doctores, 06720 Mexico City, Mexico; 2 Servicio de Endocrinología, Hospital de Especialidades Centro Médico Nacional Siglo XXI, Instituto Mexicano del Seguro Social, Cuauhtémoc 330, Doctores, 06720 Mexico City, Mexico

**Keywords:** Metformin, Sodium-glucose transporter 2 inhibitors, Prediabetes, Obesity, Morbid, Type 2 Diabetes Mellitus

## Abstract

**Background:**

Mexico has one of the highest prevalence rates of obesity worldwide. New pharmacological strategies that focus on people with class III obesity are required. Metformin and dapagliflozin are two drugs approved for the treatment of diabetes. Beyond its effects on glucose, metformin has been suggested by some studies to result in weight loss. Therapy with dapagliflozin is associated with a mild but sustained weight loss in patients with diabetes.

The primary outcome of the study is to determine if the combined treatment with dapagliflozin and metformin is more effective than monotherapy with metformin for weight loss in patients with class III obesity and prediabetes or diabetes who are awaiting bariatric surgery (including those patients who do have surgery). We also aimed to assess the effect of this combined treatment on waist circumference, triglycerides, blood pressure, and inflammatory cytokines.

**Methods:**

This randomized phase IV clinical trial will include patients with diabetes or prediabetes who are between the ages of 18 and 60 years and exhibit grade III obesity (defined as body mass index ≥ 40 kg/m^2^). Patients using insulin will be excluded. Subjects will be randomized to one of two groups as follows: 1) metformin tablets 850 mg PO bid or 2) metformin tablets 850 mg PO bid plus dapagliflozin tablets 10 mg PO qd. The sample size required is 108 patients, which allows for a 20% dropout rate: 54 patients in the metformin group and 54 in the metformin/dapagliflozin group. All participants will receive personalized nutritional advice during the study. A run-in period of one month will be used to assess tolerance and adherence to treatment regimens. Anthropometric and biochemical variables will be recorded at baseline and at 1, 3, 6, and 12 months. A serum sample to determine glucagon, ghrelin, adiponectin, resistin, interleukin 6, and interleukin 10 will be collected at baseline and before surgery, or at 12 months (whatever happens first).

Adherence to treatment and adverse and secondary events will be recorded throughout the study. An intention-to-treat analysis will be used.

**Discussion:**

Forty-six percent of the patients in our Obesity Clinic have been diagnosed with prediabetes (32%) or diabetes (14%). The use of dapagliflozin in this population could improve weight loss and other cardiovascular factors. This effect could be translated into less time before undergoing bariatric surgery and better control of associated comorbidities.

**Trial registration:**

Clinicaltrials.gov, ID: NCT03968224. Retrospectively registered on May 29, 2019.

## Background

Dapagliflozin is a selective sodium-glucose cotransporter type 2 inhibitor (iSGLT2) that blocks glucose resorption in the proximal tubule of the kidney, thereby increasing urinary glucose excretion and reducing blood glucose levels. It is currently indicated in the management of patients with type 2 diabetes (T2D) because of its sustained effect on the reduction of glycemia and glycated hemoglobin (HbA1c) [1]. As described by Baker et al. in their systematic review and meta-analysis, dapagliflozin also decreases systolic blood pressure by 4–5 mmHg and results in a 1.7 kg weight loss (95% CI 1.33 to 2.11) [2]. Furthermore, Jayawardene et al. [3] reported that dapagliflozin increases high-density lipoprotein cholesterol (HDL-c) concentrations by 1.8% to 4.4% and decreases triglyceride concentrations by 2.4% to 6.2%. Dapagliflozin effects in patients with prediabetes and other types of diabetes are currently the subject of intense research [4]. Current meta-analyses report that its adverse effects include an increase in the risk of genital tract infections (OR 3.48, 95% CI 2.33 to 5.20) [5] and a low risk of euglycemic ketoacidosis [6], among others. Most of these adverse effects are mild and do not require drug withdrawal.

Metformin is a biguanide that acts via both adenosine monophosphate-activated protein kinase (AMPK)-dependent and AMPK-independent mechanisms, inhibiting enzymes related to gluconeogenesis and lipogenesis, while also having antineoplastic effects, delaying the aging process, and regulating gut microbiota [7]. Besides being the most common drug for T2D treatment, it has been reported by some studies as having effects on weight loss. Moguel et al. prescribed metformin for a year at doses of 1500 mg/day to nondiabetic women with a body mass index (BMI) of 25 to 32.9 kg/m^2^ (group 1) and doses of 2000 mg/day to women with a BMI 33 to 41.7 kg/m^2^ (group 2) in addition to a hypocaloric, carbohydrate-modified diet. They observed that the first group decreased 8.06 ± 0.96 kg (*p* < 0.001), and the second group decreased 15.1 ± 3.3 kg (*p* = 0.011) [8].

In murine models, the glycosuria effect of dapagliflozin has been associated with a caloric deficit between 80 to 340 kcal/day [9]. As monotherapy in patients with T2D, weight loss ranges from 2 to 3 kg; meanwhile, in a 104-week, randomized, double-blinded study of dapagliflozin versus glipizide as add-on therapies to metformin, weight reductions of up to 5.1 kg (95% CI -5.7 to -4.4 kg) without regain (at least for 2 years) have been reported [10]. Other studies have shown that dapagliflozin is as effective as metformin for weight loss. In a 12-week, randomized, parallel-group, double-blind, placebo-controlled study, patients assigned to dapagliflozin 10 mg daily presented a 2.7% reduction in body weight (95%IC -3.5 to -1.8%) as compared to a weight loss of 1.7% in those assigned to the metformin group (95%CI -2.4 to -0.9%) and 1.2% in patients assigned to placebo (-2.0 to -0.4%) [11]. In a 48-week, randomized, double-blinded, placebo-controlled, parallel-group study, when used in combination with pioglitazone, dapagliflozin resulted in a 2.3 kg weight loss (95% CI -3.37 to -1.23 kg) [12].

In a systematic review by Orme et al., in patients previously treated with sulfonylurea, dapagliflozin induced a 1.54 kg weight loss (95%CI -2.16 to -0.92 kg), compared to patients treated with GLP-1 (loss: -0.65 kg, 95% CI -1.37 to 0.07 kg) and a DPP4 inhibitor (gain of 0.57 kg, 95% CI 0.09 to 1.06 kg) [13]. Furthermore, the addition of dapagliflozin in patients already treated with metformin and sitagliptin resulted in a 2.1 kg reduction (95% CI -3.2 to -1 kg) compared to placebo [14]. Finally, Bolinder et al. found that the addition of 10 mg dapagliflozin to patients inadequately controlled with metformin resulted in a decrease of 2.08 kg of total weight (-2.8 to -1.31 kg, *p* < 0.001) and decreased waist circumference by 1.52 cm (2.74 to 0.31 cm, *p* = 0.014) after 24 weeks of treatment in a randomized, parallel-group, double-blind, placebo-controlled study [15].

In a meta-analysis, Zhang et al. found that the use of dapagliflozin resulted in 20% less weight reduction than expected (2.5 kg instead of 3.05 kg) [16]. This could be related to the increase in appetite observed in DIO (diet-induced obesity) rats by Devenny et al. [7]. The effect of dapagliflozin in appetite has not been evaluated in humans.

Notably, all studies have been conducted in patients with T2D and a BMI under 40 kg/m^2^, so the effects dapagliflozin on weight and other metabolic factors have not been studied in populations with extreme obesity. Furthermore, the effects of dapagliflozin on the cytokine profile and on appetite markers like ghrelin have not been studied in this population.

Patients under evaluation for bariatric surgery have been suggested to decrease 10% of their excess weight to improve the control of some of the comorbidities that are associated with a higher surgical risk such as diabetes, hypertension, or sleep apnea [17]. This also allows evaluating the patients’ adherence to medical therapy and their capability to follow a diet. International guidelines suggest the use of drugs for weight loss when BMI is higher than 30 kg/m^2^, but only few have been approved due to their adverse effects, including increased blood pressure, anxiety, tachycardia and psychiatric effects [18]. Patients with severe obesity use a daily average of 4.4 ± 4.1 medications to control comorbidities such as diabetes, including psychoanaleptics and agents acting on the renin-angiotensin system (12%, 11.3%, and 8.2% of all medications, respectively) [19]. Therefore, most patients may not be candidates for some of these weight loss drugs, which will limit their chances to undergo surgery. SGLT-2 inhibitors have gained a privileged position in the management of T2D in the last few years due to their ability to control glucose as well as other cardiovascular risk factors including blood pressure, weight, and the lipid profile. Currently, diabetes guidelines place these inhibitors as the first choice for patients with diabetes and high cardiovascular risk [20].

The aim of the study is to determine if combined treatment with dapagliflozin and metformin (D/M) is more effective than monotherapy with metformin (M) for weight loss in patients with class III obesity and prediabetes or diabetes who are waiting for bariatric surgery.

## Methods

### Study hypotheses

The main hypothesis of the study is that dapagliflozin 10 mg tablets PO qd in combination with metformin 850 mg tablets PO bid (D/M group) in comparison with metformin alone in doses of 850 mg PO bid (M group) is associated with more weight loss for a maximum of 12 months of treatment (weight will be evaluated at months 1, 3, 6, and 12) in patients with prediabetes or T2D and class III obesity who are awaiting bariatric surgery. An exploratory hypothesis is that the combination of D/M will also be associated with a decrease in the concentration of pro-inflammatory cytokines such as interleukin-6 (IL-6) and resistin and an increase in anti-inflammatory cytokines such as interleukin-10 (IL-10) and adiponectin, as well as increases in serum insulin, glucagon, and ghrelin concentrations in comparison with M treatment. The secondary hypotheses are that patients in combination therapy with D/M will have greater reductions in BMI, systolic blood pressure (SBP) and diastolic blood pressure (DBP), waist circumference (WC), insulin resistance (IR) measured by HOMA-IR, and serum triglycerides (TG) levels in comparison with patients in the M group.

### Primary objective

The primary objective of the study is to assess if combined treatment with D/M is more effective than metformin for weight loss in patients with diabetes and prediabetes and class III obesity who are awaiting bariatric surgery at 12 months (including those patients who do have surgery after the required weight loss).

### Secondary objective

An exploratory objective of the study is to assess if the combination therapy with D/M is associated with a change in the concentration of pro-inflammatory cytokines such as IL-6 and resistin or in anti-inflammatory cytokines like IL-10 and adiponectin, as well as ghrelin, insulin and glucagon concentrations. Secondary objectives include comparisons among groups of treatment on SBP, DBP, WC, IR, and TG. We also aim to assess the prevalence of adverse effects associated with dapagliflozin use and if participants in both groups decrease the number of medications needed for the control of comorbidities.

### Type of study

This study is an investigator-initiated, controlled, parallel group, randomized controlled trial. The investigators and clinical staff involved in the care of the patients are not blinded. The investigators that register the information into data sheets and those that perform the statistical analysis will be blinded to the treatment provided.

### Study population

Patients with class III obesity (defined as a BMI greater than 40 kg/m^2^) who present with T2D or prediabetes at the time of screening or are diagnosed with these conditions during screening will be included. Diagnosis will be established according to the most recent American Diabetes Association (ADA) criteria [21]. The participating patients must have been referred for bariatric surgery to The Obesity Clinic at the Hospital de Especialidades Centro Medico Nacional Siglo XXI. The study period will be from August 2018 to August 2020.

### Study design

**Fig. 1:**
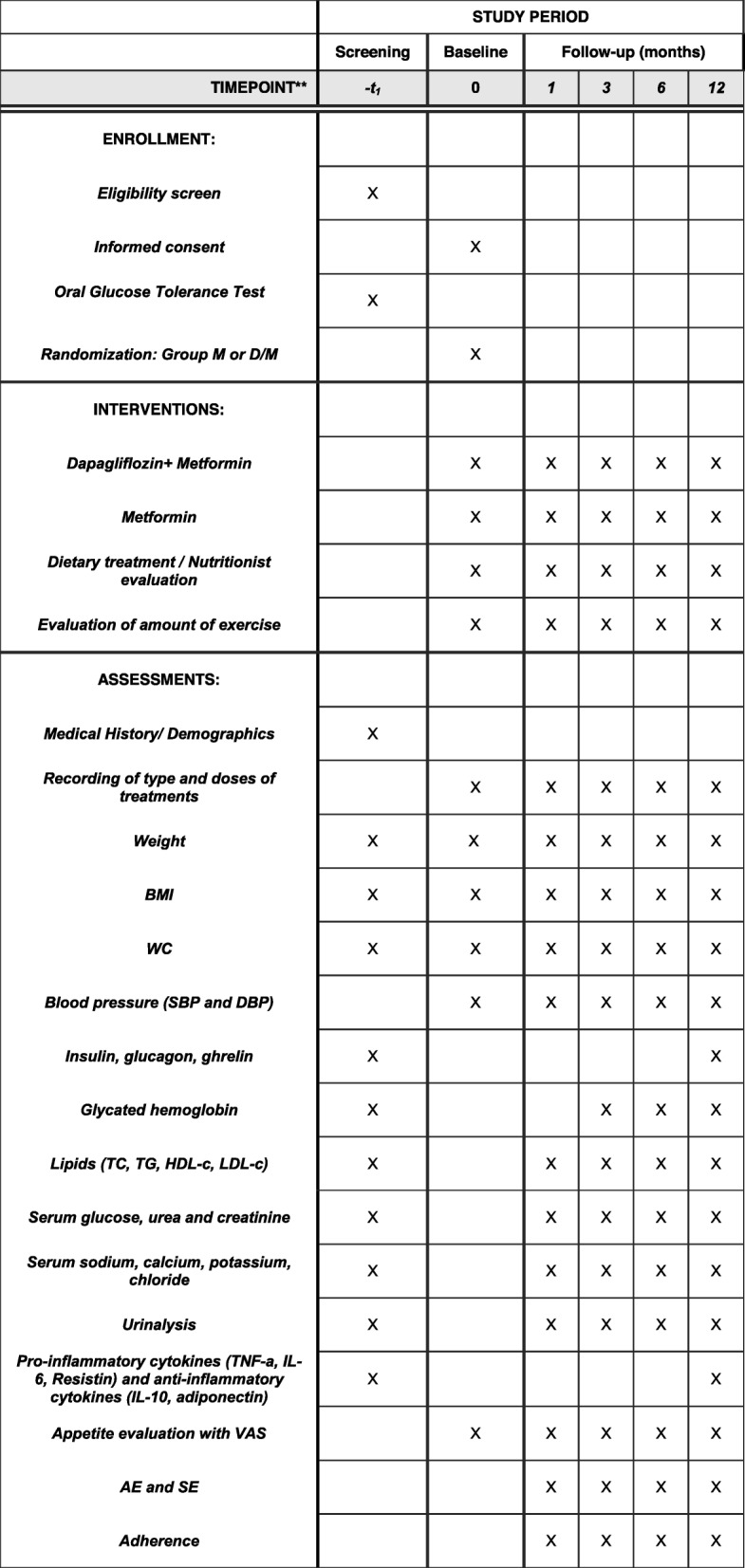
Schedule of enrollment, interventions, and assessments in accordance with Standard Protocol Items: Recommendations for Interventional Trials (SPIRIT). *M* metformin, *D/M* dapagliflozin/metformin, *BMI* body mass index, *WC* waist circumference, *TC* total cholesterol, *TG* triglycerides, *HDL-c* high density cholesterol, *LDL-c* low density cholesterol, *IL-6* interleukin-6, *IL-10* interleukin-10, *AE* adverse event, *SE* secondary event

A convenience sampling will be done for patients previously or currently diagnosed with T2D or prediabetes according to the ADA criteria, who have been referred to the Obesity Clinic of the Hospital de Especialidades Centro Médico Siglo XXI, an academic hospital in Mexico City, Mexico. The schedule of enrollment, interventions, and assessments are in accordance with the Standard Protocol Items: Recommendations for Interventional Trials (SPIRIT) Statement as outlined in Fig. 1 [22]. A checklist is also available as supplementary information (Additional file 1).

Patients will be approached and invited to participate in the study when they attend their first clinical appointment (screening visit). At this visit, we will determine an HbA1c in those patients with a previous diagnosis of prediabetes or diabetes to further assess if they qualify for the study. In those patients without previous diagnosis, we will perform an oral glucose tolerance test to determine if they have diabetes or prediabetes.

A full explanation of the purposes of the research will be provided during the screening visit. If the patients agree to participate, the informed consent will be obtained during the baseline visit by one of the investigators. During the baseline visit (a week after screening visit), the investigators will assess patient eligibility against the study inclusion/exclusion criteria. The presence of comorbidities will also be recorded at the baseline visit. The type and dose of drugs used for patient treatment will be recorded at baseline and during follow-up visits.

APGM will generate the allocation sequence for randomization using the computer software Research Randomizer [23]. Additionally, in order to ensure that the groups are comparable, six balanced blocks of participants (18 participants per block, three blocks of participants treated with dapagliflozin, and three blocks of participants treated with metformin and dapagliflozin) will be established. Once randomized, DMA and EEC will enroll participants and will assign them to either group: 1) metformin (M; 1700 mg/day) or 2) metformin (1700 mg/day) and dapagliflozin 10 mg/day (D/M).

Once assigned to the corresponding group, participants will receive an identification code that will be retained throughout the study. The investigators who will perform the data analysis will be blinded to the treatment that the participant is receiving. The investigators will analyze the data at the end of the study. Unblinding is not permissible for investigators involved in the data management. The tolerance to treatments will be assessed a month after the baseline visit. The participants will not be excluded even if they cannot tolerate treatments. If participants want to withdraw from their assigned treatment, they will be invited to continue with nutritional advice for weight loss and with other type of treatment for glucose control (e.g., use of thiazolidinediones, sulfonylureas, or insulin). This information will also be analyzed.

Anthropometric and biochemical variables will be recorded at baseline visit and at follow-up visits: 1, 3, 6, and 12 months. The determination of insulin, glucagon, ghrelin, adiponectin, resistin, IL-6, and IL-10 will be performed at the baseline visit, before surgery, or at 12 months. We aim to explore if those hormones and inflammatory markers change due to weight loss in comparison with those that do not achieve the desired weight loss at 12 months.

Registration of adverse and secondary events will take place from baseline visit and during follow-up visits. For the primary objective of the study, the data will be analyzed at 1, 3, 6, or 12 months. The amount of weight loss will be evaluated until 12 months even if participants undergo bariatric surgery or if they cannot decrease weight in the specified time. Data from participants who because of poor glycemic control require treatment with insulin or sulfonylurea will be analyzed during follow-up visits at 1, 3, 6, and 12 months as the whole group (intention-to-treat analysis). Participants who did not achieve weight loss during the one-year follow-up will be discharged to their primary care hospital for continuation with dietary treatment as recommended by local guidelines.

### Selection criteria

Inclusion and exclusion criteria are presented in Table 1. Both patients previously diagnosed with T2D or prediabetes and those detected by an oral glucose tolerance test (OGTT) at the screening visit will be included if their HbA1c is between 5.7% and 9%. Patients will be required to suspend any other treatment for glycemic control 8 h before labs and randomization to minimize the impact of such treatment on the inflammatory profile. Those participants with a lack of adherence to medical treatment (defined as consumption of less than 90% of tablets granted) or less than 80% of adherence to the nutritional recommendations or who miss more than 20% of the clinical appointments; those with an adverse effect grade 4 or 5 (assessed with the Common Terminology Criteria for Adverse Effects, CTCAE v 5.0 [24]; those with intolerance to metformin or dapagliflozin evaluated during the run-in period; and those that initiate any other treatment or product for weight loss despite being advised against it will also be considered for analysis. Those participants with genital or urinary tract infection (UTI, detected by symptomatology and evidenced by urinalysis) at the screening visit will be treated with proper antibiotic treatment and invited to participate a week later if the infection is resolved as demonstrated by a new urinalysis.

**Table 1:** Inclusion and exclusion criteria

Inclusion criteria	Exclusion criteria	Withdrawal criteria
Age between 18 and 60 years	Current use of insulin or SU	Becoming pregnant during the study
BMI ≥ 40 kg/m^2^	Chronic kidney failure with eGFR ≤ 60 ml/min/1.73 m^2^	Tobacco use (in the last month) (for the secondary objectives)
HbA1c < 9%	Use of loop diuretics	Use of steroids drugs during the study (for the secondary objectives)
Patients willing to participate and that sign the informed consent letter	Concomitant illnesses that predispose to volume depletion, weight loss (including oncological diseases), or metabolic acidosis (including drug users)	
Candidates to bariatric surgery	History of recurrent genital or urinary tract infections	
	Current treatment with medications/products for weight control	
	Untreated or uncontrolled hypothyroidism (defined through TSH higher than 4.2 μU/ml)	
	Pregnant or lactating mother	
	Tobacco use (in the last month)	
	Current use of steroids or nonsteroidal anti-inflammatory drugs	

*BMI* body mass index, *HbA1c* glycated hemoglobin, *SU* sulfonylureas, *eGFR* estimated glomerular filtration rate, *TSH* thyroid stimulating hormone, *CTCAE* Common Terminology Criteria for Adverse Effects

### Nutritional, anthropometrical, and biochemical assessment

A single nutritionist from the Nutrition Department will evaluate all participants throughout the study. Each participant receives an individualized diet depending on initial weight, ideal weight, and dietary patterns at the time of assessment. We do not expect dietary advice to influence our results because 1) the same nutritionist will assess all the participants, and 2) participants will be randomized. We will analyze data from those participants with lack of adherence to diet (defined as compliance of at least 80% of indicated diet) for the intention to treat analysis. The diet compliance will be assessed with concordance between the indicated foods (according to recommended kcals) and those reported by the participant using a 24-h recall with portion size estimation aids; these data will be collected for 3 days, including Sundays [25].

The nutritionist will determine the amount of aerobic exercise in minutes per week in baseline and follow-up visits. Participants are required to reach a minimum of 150 min per week of aerobic exercise during the baseline visit. The exercises are indicated specially for overweight people with limited mobility, and they can be performed during the day, with no special equipment required. The amount of exercise will be evaluated using a seven 24-h physical activity record, a week before each appointment [25]. Exercise will not be considered an inclusion criterion, but it will be evaluated post hoc as a confounding variable.

At the baseline and follow-up visits (months 1, 3, 6, and 12), the nutritionist will measure hunger through a visual analog scale (VAS). The participants will answer the question: How hungry do you feel? by making a mark on a 100 mm straight line, where the two extreme answers (not hungry or hungry) are anchored on opposite ends of the line [25]. Participants will be required to answer the VAS after being fed. For this purpose, we ask the participants to eat the breakfast recommended by the nutritionist.

A certified anthropometrist (certified by the International Society for the Advancement of Kinanthropometry) will evaluate weight using a scale with maximum weight capacity of 300 kg (662 lb) and precision of 100 g (0.22 lb); height using a fixed stadiometer; WC using a fiberglass tape measure according to procedure manual for nutrition projects of the Center of Research in Nutrition and Health of the National Institute of Public Health of Mexico [26], and the procedure manual for assessment for clinic and anthropometric measures in adults and older adults of the Mexican Ministry of Health [27]. BMI will be calculated at screening, baseline, and follow-up visits. The percentage of excess body weight loss will be calculated using the following formula: %EBWL = Initial BMI – Final BMI/Initial BMI – Ideal BMI * 100, and the percentage of weight loss will be calculated according to %WL = Initial weight – Final weight/Initial weight * 100 in the follow-up visits. A trained nurse will evaluate the SBP and DBP by considering the mean average of two determinations taken 5 min apart, according to the procedure manual for assessment for clinic and anthropometric measures in adults and older adults of the Mexican Ministry of Health [27].

The following laboratory determinations will be performed at the screening visit and the follow-up visits at 1, 3, 6, and 12 months: glucose, total cholesterol (TC), HDL-c, low-density lipoprotein cholesterol (LDL-c), TG, urea, creatinine, serum sodium, calcium, potassium and chloride, and urine analysis. HbA1c will be evaluated at the screening visit and at 3, 6, and 12 months. The measurement of insulin, glucagon, ghrelin, adiponectin, resistin, IL-6, and IL-10 will be performed at the baseline visit and at the appointment prior to surgery (depending on the response of each participant) or after a year (if weight loss is not achieved) using ELISA (enzyme linked immunosorbent assay).

### Evaluation of adherence and adverse effects

One of the researchers not directly involved in the care of participating patients will provide the tablets needed for daily intake for a month and then the number of tablets required for visits in a sealed envelope. Adherence to medical treatment will be evaluated in follow-up visits after the run-in period. The participants will be asked to return their envelope and must consume at least 90% of the pills.

The adverse and secondary events will be recorded from the baseline visit and throughout the follow-up visits. Each adverse event will be evaluated by all the researchers and classified according to CTCAE version 5. Accordingly, a grade 4 or 5 adverse event indicates discontinuation of treatment; grade 3 must be submitted to review by the medical team, and grades 1 or 2 require registration and intervention but not discontinuation of treatment. Adverse events grade 4 or 5 will be immediately reported to the ethics committee for follow-up and recommendations.

### Sample size

Weight loss after dapagliflozin treatment has not been previously used as primary outcome in randomized controlled trials (RCT) of patients with prediabetes or diabetes and grade III obesity. Zhang et al. performed a meta-analysis that included seven RCTs evaluating the effect of metformin combined with SGLT2-inhibitors (dapagliflozin 10 mg/day, canagliflozin 300 mg/day, empagliflozin 25 mg/day, or ipragliflozin 300 mg/day) vs. metformin (at doses of 1.5 to 3 g/day) with placebo on HbA1c; fasting plasma glucose; and body weight over 24 weeks, 1 year, and 2 years. The total sample was 2847 patients with a mean BMI of 31.7 ± 4.9 kg/m^2^. After a year of treatment, the placebo–metformin control group showed a reduction in body weight of 1.1 ± 3.39 kg, whereas the SGLT-2 inhibitor–metformin group showed a 3.6 ± 4.22 kg reduction with a mean difference of − 2.6 kg (− 3.17 to − 2.03 kg), *p* < 0.00001 [16]. Using these data, we calculated a sample size using a mean difference formula. With a power of 80% (alpha level of 0.05) in a two-sided T-test, the sample size required is 90 patients: 45 patients in the M group and 45 patients in the D/M group. The final sample size required is 108 patients: 54 patients in metformin and 54 patients in the dapagliflozin group, allowing for a 20% dropout rate.

### Statistical analysis

Quantitative variables will be presented as means and standard deviation or medians with interquartile ranges according to the data distribution. Categorical variables will be presented as frequencies or percentages. Intention-to-treat analyses will be applied as the primary analysis. The primary endpoint (weight loss at 12 months) will be analyzed using analysis of covariance (ANCOVA) while adjusting for baseline weight. We will also use a mixed-model regression analysis to estimate the treatment effect at 1, 3, 6, and 12 months in other quantitative variables (due to the possibility of missing values), while considering as fixed effects the assigned treatment, sex, prediabetes or diabetes status, and random effects as the variability in the weight loss through the time. For qualitative variables analysis, we will use a mixed logistic regression analysis. A two-tailed *p* value < 0.05 is considered statistically significant. Statistical analysis will be performed using statistical packages: Statistical Package for the Social Sciences (SPSS) version 17.0 and STATA version 11.0.

### Trial management group, steering committee, monitoring, and data collection

The trial management group consists of MM, AFH, CRR, and MAMA, and it is responsible of the design, data analysis, and publication of the trial. The primary investigators are AFH and MM.

Data will be collected and stored in properly codified individual files (VMZ, EEC, DMA, and APGM). Those documents are stored at the experimental sites in accordance with the rules and regulations of the Mexican Institute of Social Security (IMSS, Instituto Mexicano del Seguro Social). The study is monitored by the National Commission for Scientific Research (CNIC: Comisión Nacional de Investigación) of the IMSS. The National Commission of Scientific Research acts as a Trial Steering Committee. It is integrated by independent clinical and basic investigators (not affiliated to the same hospital or research center related with the investigators) that provide overall supervision and ensure that all registered trials throughout Mexico are conducted with rigorous standards. Those investigators are anonymous and randomly selected, and the evaluation process is through an electronic platform. A technical report informing the progress of the study must be presented to The National Commission for Scientific Research.

The Data Monitoring Committee consists of two investigators with expertise in methodology and statistics. They will receive interim reports and data summaries during the trial, monitor the data quality and participant safety, and assume responsibility for stopping the trial if necessary. The evaluations will be conducted every 6 months.

### Publication and diffusion of results

The results of the study will be submitted to international peer-reviewed journals, irrespective of its positive, negative, or inconclusive outcomes. Furthermore, the results will be presented at scientific conferences as abstracts, oral presentations, and posters.

The datasets generated and analyzed during the current study will be available from the corresponding author on reasonable request.

## Discussion

Mexico has one of the highest prevalence rates of overweight and obesity worldwide. According to the National Health and Nutrition Survey, Mid Step (ENSANUT MC: Encuesta Nacional de Salud y Nutrición de Medio Camino 2016), 33.3% of Mexicans older than 20 years are obese, with a prevalence of 27.7% in males and 38.6% in females. This survey also showed that the prevalence of class III obesity in males was 1.7% and 4.1% in females (2.4-fold higher in the latter) [28].

In 2008, a multidisciplinary clinic for the treatment of severe obesity and its related comorbidities was inaugurated at Hospital de Especialidades of Centro Médico Nacional Siglo XXI. One of the purposes of the Clinic is to offer bariatric surgery to qualifying patients, who are defined as those who achieve an appropriate weight loss (EBWL of 10%). Several studies have proven that weight reduction prior to surgery decreases surgery-related complications and decreases hospitalization time [17]. Unfortunately, as previously observed in a quasi-experimental study performed at our Obesity Clinic, 46% of our patients do not achieve the required weight loss after a year of dietary treatment. Furthermore, 46% of our patients have prediabetes or T2D, 66% are hypertensive, and 33% have dyslipidemia [29]. T2D treatments available in our clinic include metformin, pioglitazone, acarbose, and insulin (Neutral Protamine Hagedorn insulin, glargine and ultra-rapid insulin analogs). The patients with diabetes and obesity could benefit from the use of weight loss pharmacological treatment as recommended in 2015 by the Endocrine and Obesity Society [18]. However, Mexico’s Federal Commission for the Protection against Sanitary Risks (COFEPRIS: Comisión Federal para la Protección contra Riesgos Sanitarios) has approved only two treatments: orlistat and liraglutide [30]. The former has a low adherence rate associated to its secondary effects, and the latter is not covered by social security due to its high cost. Therefore, we propose the use of a low-cost and secure treatment such as dapagliflozin for weight reduction in patients with grade III obesity and T2D or prediabetes. In our institution, the most common dose of metformin prescribed for glucose control is 850 mg bid [31]. To assess if dapagliflozin has a higher efficacy, we decided to compare its addition to the usual treatment instead of using combination therapies such as Xigduo, which contains higher amounts of metformin (dapagliflozin 5 mg with metformin 1000 mg). Nowadays, both treatments are only indicated for glucose lowering in patients with diabetes or prediabetes. However, we aim to probe that beyond this effect, antihyperglycemic drugs such as metformin and dapagliflozin could also decrease weight in patients with higher grades of obesity.

Several studies have reported that metformin induces a modest decrease or no change, in weight [32, 33]. In an open randomized cross-over study, Hermann et al. reported a 2.64 kg loss of total weight (95% CI -4.23 to -1.05 kg) after one year of treatment with metformin (patients with an average weight of 76.4 kg) [34]. Similarly, in a quasi-experimental study, Stumvoll et al. observed a total weight loss of 2.7 ± 1.3 kg in a study conducted in 10 T2D patients with an average BMI of 32.1 ± 3.2 kg/m^2^ and treated with metformin at a dose of 2550 mg (850 mg three times a day) [35]. In addition, several studies have demonstrated that dapagliflozin effectively induces weight loss, decreases WC, reduces systolic and diastolic blood pressure as well as TG, and increases HDL-c both as monotherapy and in combination with other antidiabetic medications [10–15]. No studies have been conducted in patients with a BMI higher than 40 kg/m^2^ to assess the weight loss capacity of the combination of D/M.

In their systematic review and meta-analyses, Zhang et al. calculated that a urinary glucose excretion of 71.2 g/24 h induced by iSGLT2 should decrease 3.05 kg. However, only a 2.5 kg weight reduction was achieved, representing 20% less than expected [16]. This result led to Devenny et al. evaluating possible compensatory mechanisms that attenuate the effect of dapagliflozin. In a group of DIO rats, orally administered dapagliflozin in different doses induced weight and fat mass loss in a dose-dependent way. In this study, the effect on weight and fat mass was greater in those animals with restricted food consumption. Furthermore, in the group of animals fed *ad libitum*, they observed an increase in appetite as of day 7 and throughout the study. They evaluated appetite as the amount of food consumed each day in grams multiplied by the caloric value of the diet. They concluded that the increased appetite decreases the weight loss capacity induced by dapagliflozin [9]. This conclusion has not been evaluated in humans. In our protocol, we propose to evaluate ghrelin concentration, as a surrogate indicator of appetite [36].

Additionally, few studies have evaluated the effect of dapagliflozin on the concentration of inflammatory cytokines. In a single-arm interventional study, Okamoto et al. observed that dapagliflozin administered for 12 weeks decreases highly sensitive CRP (hs-CRP) and increases adiponectin [37]. Once again, this study included patients with an average BMI of 32.7 ± 6.5 kg/m^2^. According to the effect of decreased weight and WC previously reported in patients with grade I and II obesity treated with dapagliflozin, we expect a decrease in resistin and IL-6 concentrations and an increase in IL-10 and adiponectin concentrations.

Finally, we aim to investigate if dapagliflozin treatment increases insulin and glucagon secretion in patients with obesity. Chronic hyperglycemia leads to a defect in insulin production due to glucotoxicity. The lowering of glucose by dapagliflozin has been suggested to reduce glucotoxicity and improve insulin secretion [38]. Furthermore, the normalization of blood glucose levels as well as the decrease in HbA1c levels and weight reduction induced by dapagliflozin seems to be related with an improvement in insulin sensitivity [38]. Some studies suggest that dapagliflozin could increase endogenous glucose production due to an increase in glucagon secretion by exerting a direct effect on alpha cell function [39]. Those effects remain controversial in people with obesity.

## Trial status

The study is currently recruiting and enrolling participants according to version 2 of the protocol in June 2019. Recruitment began on July 7, 2018, and the approximate date for completion of recruitment will be July 7, 2020.

## Supplementary information

**Additional file 1.** Checklist for the report of study protocols according SPIRIT.

## Data Availability

The datasets used and analyzed during the current study will be available from the corresponding author on reasonable request.

## Abbreviations

%EBWL: Percentage of excess body weight loss
%WL: Percentage of weight loss
ADA: American Diabetes Association
AMPK: Adenosine monophosphate-activated protein kinase
ANCOVA: Analysis of covariance
BMI: Body mass index
CNIC: Comisión Nacional de Investigación Científica
COFEPRIS: Comisión Federal para la Protección contra Riesgos Sanitarios
CTCAE: Common Terminology Criteria for Adverse Effects
D/M: Dapagliflozin/metformin
DBP: Diastolic blood pressure
DIO: Diet-induced obese
eGFR: estimated glomerular filtration rate
ELISA: Enzyme linked immunosorbent assay
ENSANUT: Encuesta Nacional de Salud y Nutrición
FIS: Fondo de Investigación en Salud
HbA1c: Glycated hemoglobin
HDL-c: High-density lipoprotein cholesterol
hsCRP: Highly sensitive C-reactive protein
IL-10: Interleukin-10
IL-6: Interleukin-6
IMSS: Instituto Mexicano del Seguro Social
IR: Insulin resistance
iSGLT2: Sodium-glucose cotransporter-2 inhibitors
LDL-c: Low-density lipoprotein cholesterol
M: Metformin
OGGT: Oral glucose tolerance test
OR: Odd ratio
RCT: Randomized controlled trial
RCT: Randomized controlled trials
SBP: Systolic blood pressure
SPIRIT: Standard Protocol Items: Recommendations for Interventional Trials
SPSS: Statistical Package for the Social Sciences
SU: Sulfonylureas
T2D: Type 2 diabetes
TC: Total cholesterol
TG: Triglycerides
TSH: Thyroid stimulating hormone
UTI: Urinary tract infections
VAS: Visual analog scale
WC: Waist circumference
